# Resilience of Hmong Older Adults: Coping With Loneliness

**DOI:** 10.1093/geroni/igaa057.1026

**Published:** 2020-12-16

**Authors:** Cindy Vang, Michael Sieng, Mingyang Zheng

**Affiliations:** 1 University of Minnesota, Twin Cities, Saint Paul, Minnesota, United States; 2 Arizona State University, Tempe, Arizona, United States; 3 University of Minnesota-Twin Cities, Saint Paul, Minnesota, United States

## Abstract

Older refugees are especially susceptible to loneliness with their history of trauma, forced migration, and social isolation in host countries. Rates of loneliness among older immigrants range from 24% to 50%. Despite their heightened vulnerability to loneliness, coping mechanisms among this population remain understudied. The purpose of this study was to examine how community-dwelling Hmong older adults, an aging refugee group, cope with loneliness. The data was drawn from a larger constructivist grounded theory study aimed at understanding the loneliness experiences of community-dwelling Hmong older adults. Semi-structured individual interviews were conducted in the Hmong language with 17 Hmong age 65 and older residing in Northern California. Data was collected and analyzed in an iterative and comparative process using initial coding, focused coding, and connecting the focused codes to form categories and subcategories. Five coping mechanisms emerged from the data: (a) religious and spiritual beliefs; (b) social support; (c) wandering; (d) activity engagement; and (e) avoidance and control. Coping mechanisms utilized by Hmong older adults in this study highlighted the resilience of this aging population and the lack of culturally-relevant programs to prevent and address their persistent loneliness and emotional distress. Implications for research, practice, and policy suggests the need for greater culturally- and linguistically-competent services informed by Hmong older adults.

